# Genealogical Trees of Scientific Papers

**DOI:** 10.1371/journal.pone.0150588

**Published:** 2016-03-08

**Authors:** Michaël Charles Waumans, Hugues Bersini

**Affiliations:** École polytechnique de Bruxelles, ULB, CoDE-IRIDIA, 50, Av. F. Roosevelt, CP 194/6, B-1050 Brussels, Belgium; University of Namur, BELGIUM

## Abstract

Many results have been obtained when studying scientific papers citations databases in a network perspective. Articles can be ranked according to their current in-degree and their future popularity or citation counts can even be predicted. The dynamical properties of such networks and the observation of the time evolution of their nodes started more recently. This work adopts an evolutionary perspective and proposes an original algorithm for the construction of genealogical trees of scientific papers on the basis of their citation count evolution in time. The fitness of a paper now amounts to its in-degree growing trend and a “dying” paper will suddenly see this trend declining in time. It will give birth and be taken over by some of its most prevalent citing “offspring”. Practically, this might be used to trace the successive published milestones of a research field.

## Introduction

Among the many types of social networks that have gained a considerable attention these last years, scientific publication citation networks [1] [2] [3] are among the most popular. Two main reasons can explain this growing interest. First, they seem to present the now classical scale-free topology [4] of most of the social types of network with few papers turning hub and the large rest of them attracting much less citations in time. But above all, a better understanding of their topology [5] [6] [7] and the way they grow has become a very sensitive issue [8] [9] on account of the importance taken by citations in the evaluation of a researcher carrier and his professional progression [10] [11] [12] (in our scientific communities, it is no more “publish or perish” but “be cited or perish”). Generally these networks have been analysed in their static version: degree distribution, presence of clustering [13], discovery of the most popular papers by means of ranking algorithms [14] [15] [16] [17] [18] [19] or other similar static types of algorithm. In this paper, instead, these same networks are studied in their dynamic version i.e. the way each of their node in-degree (the number of citations this node receives) increases in time.

Using four well-known datasets, we investigate the increasing speed of the number of citations received by each of these papers. The popularity of a paper is now being centered on this in-degree increasing curve. We discuss how this new measure allows assessing the success and fate of a publication using a novel approach.

On the basis of this new characterization of publication nodes by the way their citations grow in time, it becomes equally possible to install our whole approach in a sort of evolutionary framework [20]. The “fitness” of a paper amounts to this growing trend and a “dying” paper would suddenly see this trend declining in time to be further taken over by some of its most prevalent citing “offspring”. Departing from an old but important initial paper, our proposed approach might allow any researcher to recapitulate the history of his research field by tracing the successive published milestones.

In this paper, we first describe the datasets as well as the most prominent growing trends identified. A new set of features is then discussed to better follow the gain or loss of popularity in terms of citations growing speed. We then describe an original algorithm for drawing genealogical trees departing from the loss of popularity of successful papers and evidentiating their most prevalent citing successors. Three conditions are proposed and illustrated for a paper A to be a natural successor of paper B: 1) Paper A must cite paper B—2) paper B must see its citations in-degree growth begin to decrease and 3) paper A must in contrast see its citation in-degree growing. We limit this paper to the static version of the algorithm (just delivering snapshots of any article genealogical tree) but the dynamical version is described as well without showing the associated movies that it does generate.

## Datasets

Four citation networks are used for this study: The ArXiV HEP-TH and ArXiV HEP-PH as released for the 2003 KDD Cup, the American Physical Society and PubMed [Section “Data Availability Statement”]. Both ArXiV networks possess an average of 30k nodes and 400k directed edges with temporal metadata, whereas the APS reaches the count of 450k nodes for 4.5m edges; finally, the PubMed network is considerably larger with its 6.8m articles for 23m references.

**Fig 1:**
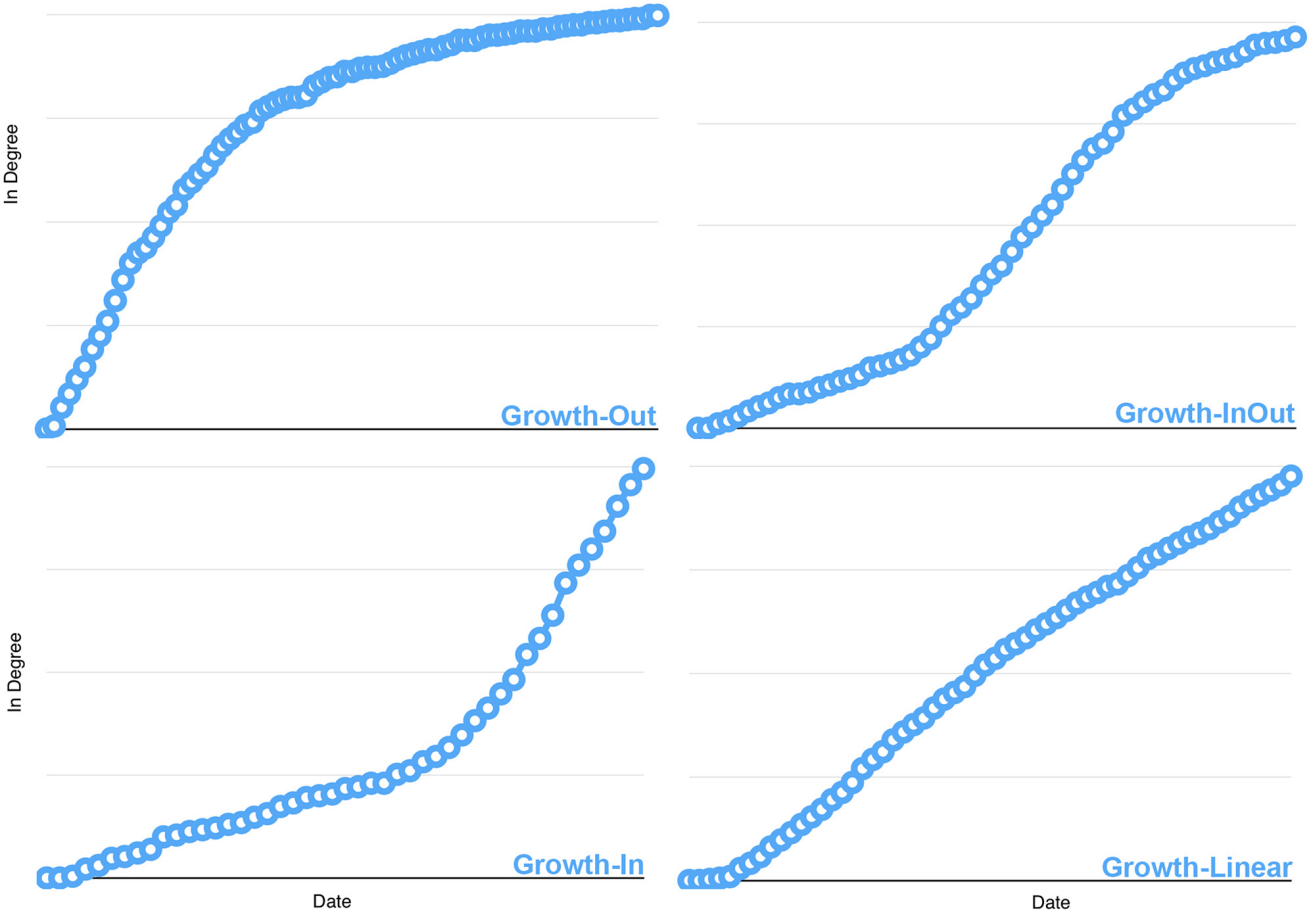
Examples of the four most representative in-degree growth trends observed in all datasets.

Following the recent developments in citation network analysis, we carefully examine the way the nodes in-degree grow in time. All articles do follow different types of evolution but they still present similarities in their growth trends. In a very first approximation, these trends can be loosely described as logarithmic, linear or exponential curves, while not exactly fitting any of these functions (See Fig 1). Articles rapidly losing citations after having received a large attention may also be seen as roughly matching a sigmoid time-evolution.

### Growth trends

Since none of the observed growth curves perfectly fits any of the afore mentioned functions, let’s rather designate them using more neutral terms that resemble those of the Robert Penner’s Tweening functions [21]. The four types do correspond to the following observations:

- **Growth-Out**: Papers presenting similarities to a logarithmic growth are losing the interest of the community. They still gather citations but less and less as time passes.
- **Growth-InOut**: This type of article starts by gathering few citations during the first months following its release, but gathers a lot more attention after a certain period of time. After this considerable gain of attention, it starts losing this initial interest. This type has its counterpart: Growth-OutIn. Both are less present among the different datasets as they reflect transient states where one article growth shifts from one category to another.
- **Growth-In**: These papers gather a lot of interest and are cited more and more as time passes (similar to an exponential growth). Although this kind of article can be considered as stars in the field, ultimately they will also lose citations over the years.
- **Growth-Linear**: The articles characterized by a linear curve are on average presenting a constant growth. As a matter of fact, almost all articles are assimilable to these Growth-Linear ones during the first months following their publication but very few keep this constant pace for long.

An article may start, at some point in time, losing interest and thus citations as time passes. The reason why this loss occurs was, as far as we know, not examined in previous works. Possible explanations are: The publication of a new article covering a possible new field of research in the steps of the previous one, or a new article by the same author, colleagues or rivals, describing an evolution of his research which is from that point in time considered to be more up-to-date to refer. Another question is: “To whom the citations an article loses apparently seem to benefit, who are the natural successors?”

We postulate that a simple and continuous observation of the incoming edges entering an article or its offspring (the successful articles that cite it) allows to trace the evolution of their genealogy, the way their popularity decreases to be replaced by more recent papers. To restrict the successors of any article to the papers citing it makes a lot of sense since citations are supposed to be a form of tribute or recognition to the papers that most influence a new development and from which it “inherits” the most. We go further by supposing that these offspring must also be responsible for the loss of attention plaguing their parents. We thus have a way to initiate the building of the trees from the star articles declining in popularity.

Being able to detect precisely when and how this transfer of citations occurs poses a challenge. Specific features should indicate not just whether an article gains or loses citations but how. A small decline of the citation rate does not specifically mean that an article is being taken over by another one, but a consequent one could. This detection consequently requires to better identify the growing dynamic regime in which an article falls at each point in time. In what follows, we propose a simple set of features allowing to better characterize the way each article in-degree citation rate changes in time.

### Growth characterization

**Fig 2:**
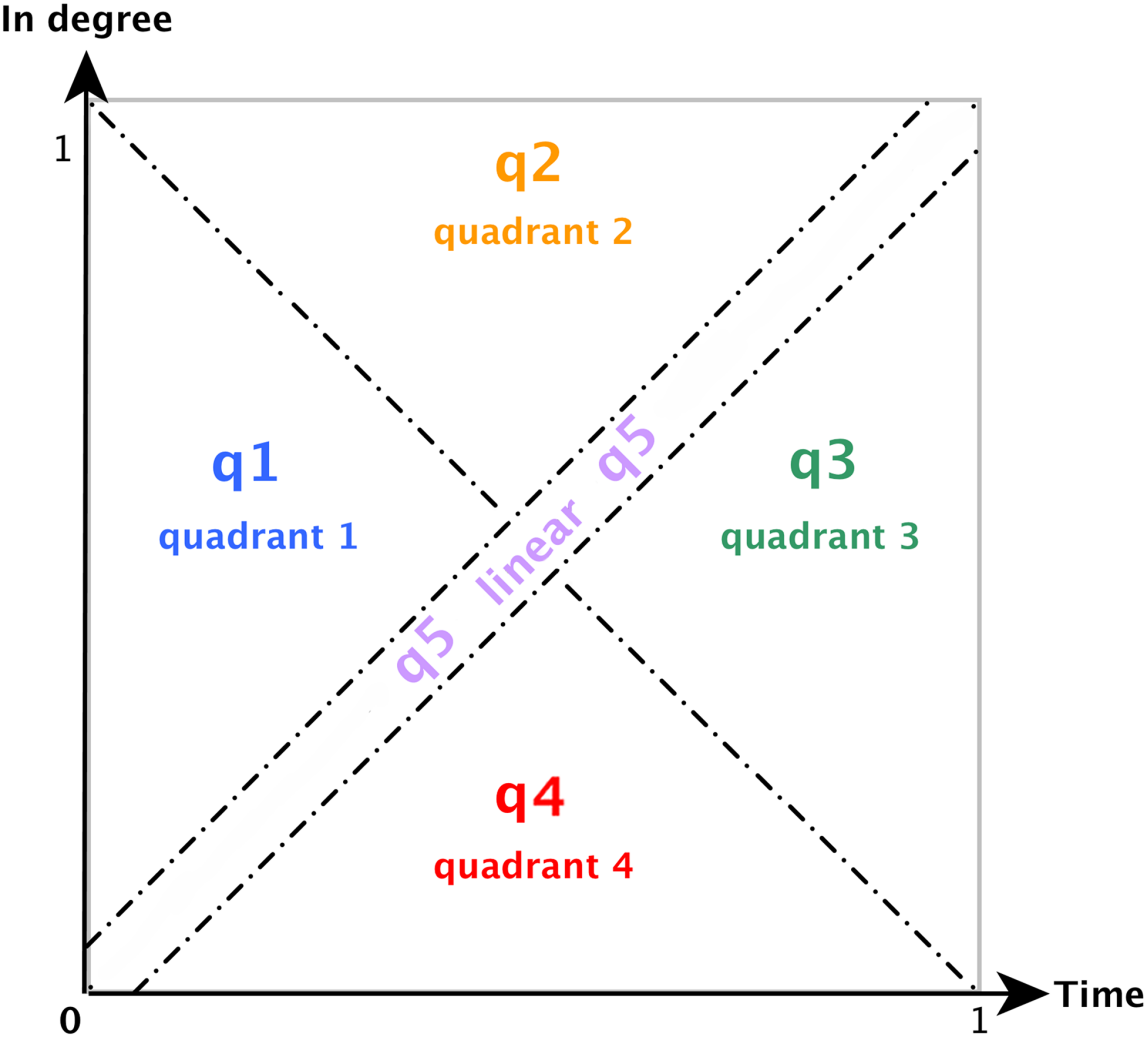
Definition of the four quadrants and the exclusion area around the diagonal, along with the naming convention used.

These features are called quadrants although they differ from the analytical geometry definition and just refer to quarters of a well-defined area. A quadrant value defines the “amount of samples from a given normalized time series present in either one of the four quadrants defined by cutting the axis (i.e: X-axis and Y-axis) in two half using the diagonals.” (Fig 2) This notion allows to clearly identify the shape of a given curve even better than a direct fitting would. More specifically, when following the evolution of a curve over time, the key transition from Growth-In to Growth-Out becomes more salient using quadrants as shown in the following examples. Different approaches were studied first to develop those features but none was as simple and efficient to detect the transitions from one dynamical regime to another.

Just using these four quadrants, Growth-Linear curves remain difficult to identify since they do oscillate among all of the four areas. In order to better identify this dynamical regime, an extra exclusion zone is added out of the quadrants. A point close enough to the diagonal going from (0,0) to (1,1) will be considered to be in this fifth “quadrant”. By resorting to these five features, all the types of curve previously observed are clearly identifiable. Besides the capacity of this method to properly identify each type of growth, it can further characterize the way these types change in time.

Next, examples of articles from the different categories of growth along with the graphical representation of their evolution in time are presented.

#### Growth-In evolution

**Fig 3:**
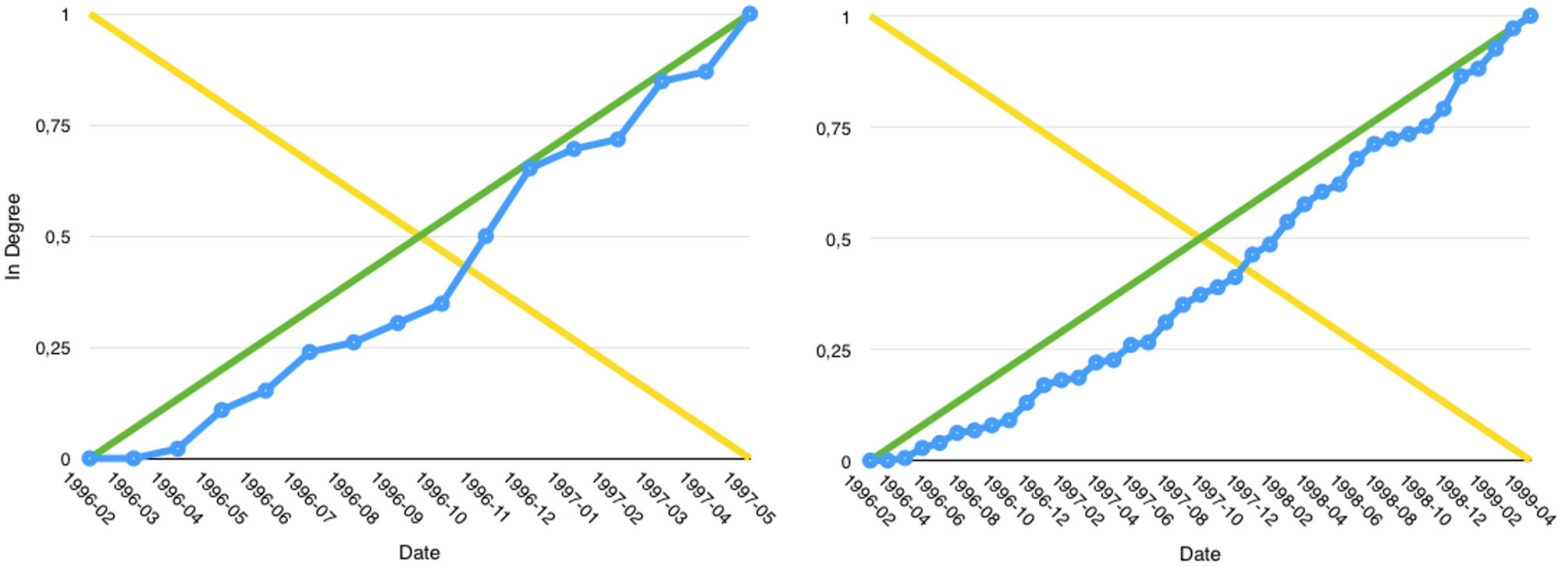
Article AU6vlHpu05SKIEIHnj3o in degree growth in 1997-06 then 1999-07.

Such articles (Fig 3) usually start as Growth-Linear during the first few months following their publication, then the Growth-In behaviour accentuates. They start by having a high *q*5 value until the *q*3 and *q*4 values greatly increase.

#### Growth-Out evolution

**Fig 4:**
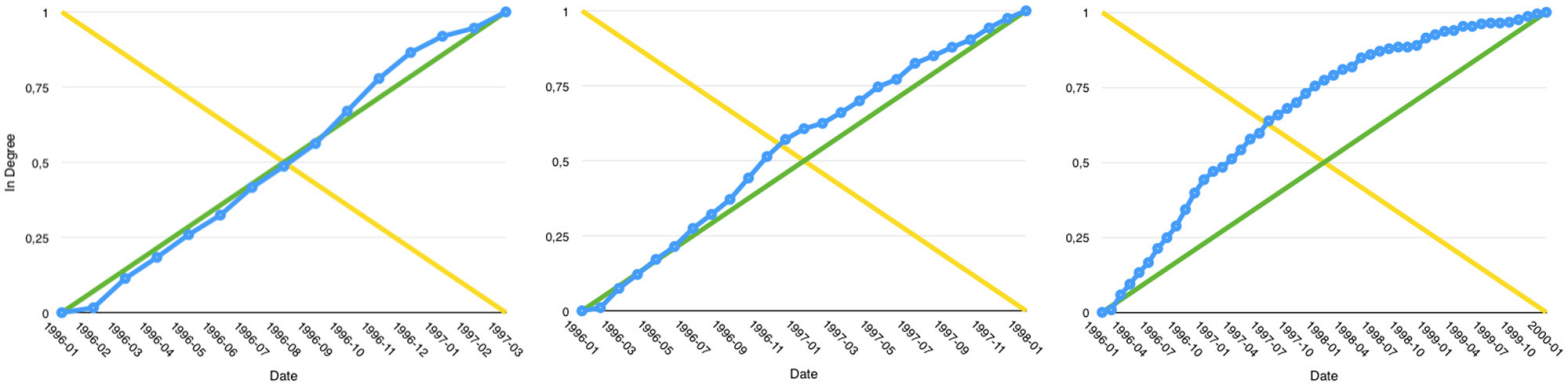
Article AU6vljQ505SKIEIHnpJC in degree growth curve in 1997-03, 1998-01 then 2000-01.

**Fig 5:**
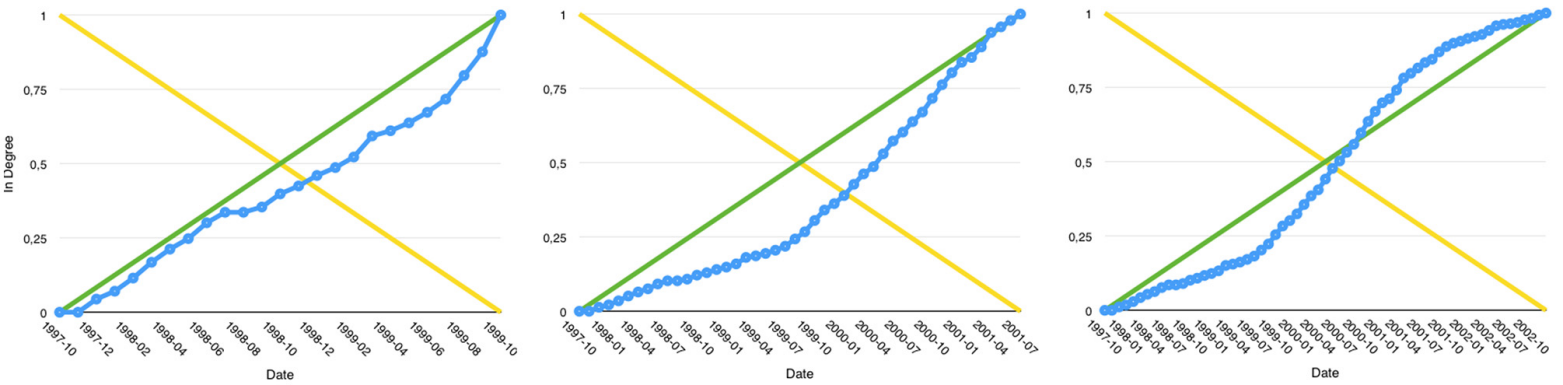
Article AU6vljQ505SKIEIHnpJC evolution. The highest quadrant value of them all indicating it’s appartenance to one of the Growth trends previously identified.

This kind of article may as well start as Growth-Linear before its Growth-Out behaviour becomes manifest. This implies a high *q*5 value up to the moment the *q*1 and *q*2 values greatly increase (Fig 4). This indicates the presence of a downturn in the growth rate. The way the transitions between those phases are observed and labelled is well illustrated in an example shown in Fig 5.

#### Growth-InOut evolution

**Fig 6:**
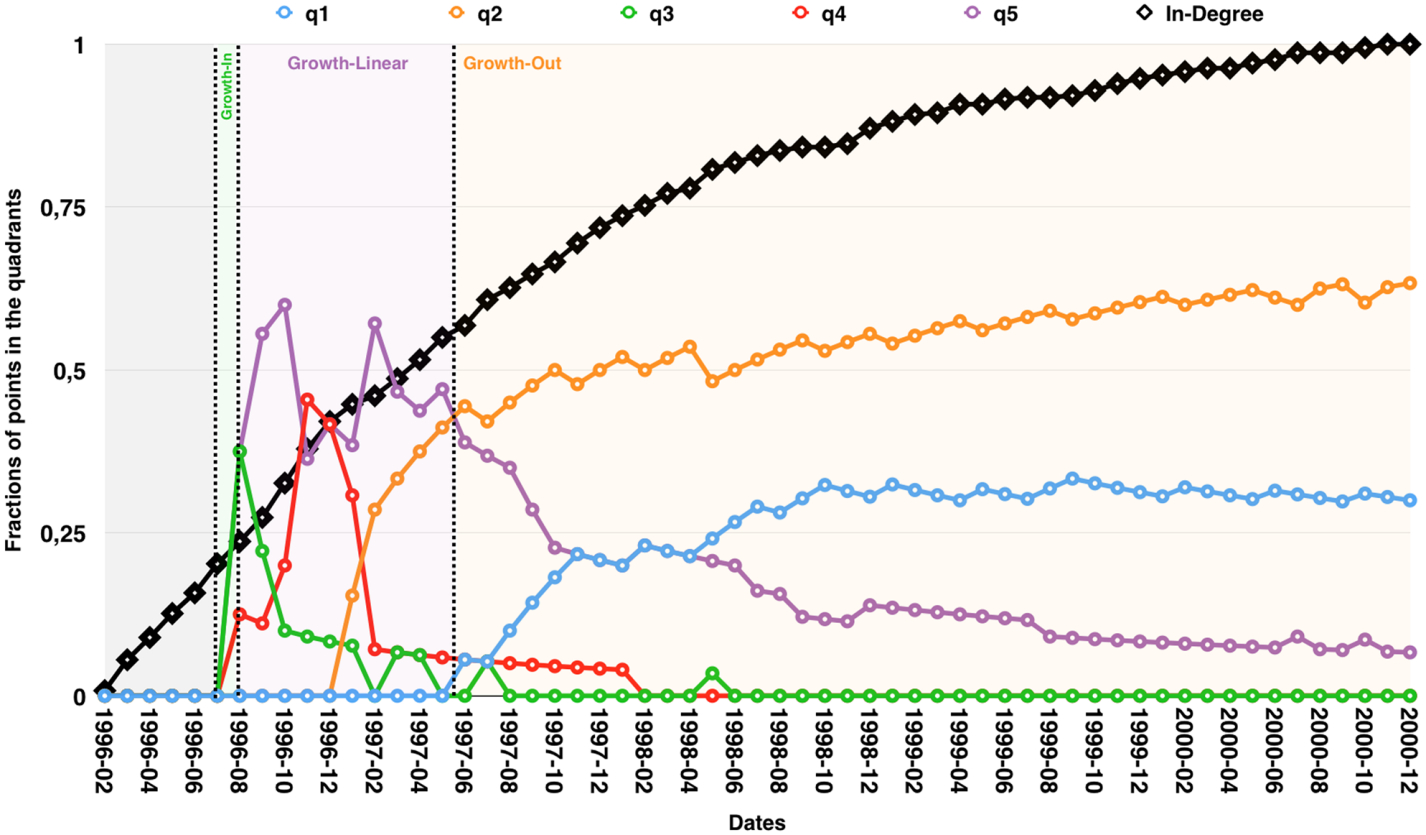
Article AU6vlW3P05SKIEIHnmbu in degree growth in 1998-10, 2001-07 then 2002-12.

Those articles, much smaller in number, are the most intriguing ones. They eventually start presenting a high *q*5 value up to attend a shift with an increase of their *q*3 and *q*4 values. When they start to suffer from a loss of popularity, the *q*3 value starts to decrease as *q*2 increases, leading to high *q*2 and *q*4 values (Fig 6).

### Change of regime

We have so far proposed stable features to characterize the growth of any given curve. They allow to identify properly Growth-Linear, Growth-In, Growth-Out as well as Growth-InOut or Growth-OutIn trends and the transitions among those types of growth over time. The Fig 5 illustrates this aspect. Looking at the evolution of the quadrant values over time, we can easily see that this specific article is first Growth-In to then become Growth-Out.

When an article is published, it may be updated even if its main content will not change. This is a particular characteristic of citations networks. It justifies why the quadrant values computation are always done using an opening time window, considering the in-degree values for each month, since the time of publication to the time chosen to make the computation. For other types of networks such as social ones, the content of a node (e.g: the feed of a person) being updated regularly, a sliding time window could be more appropriate.

**Fig 7:**
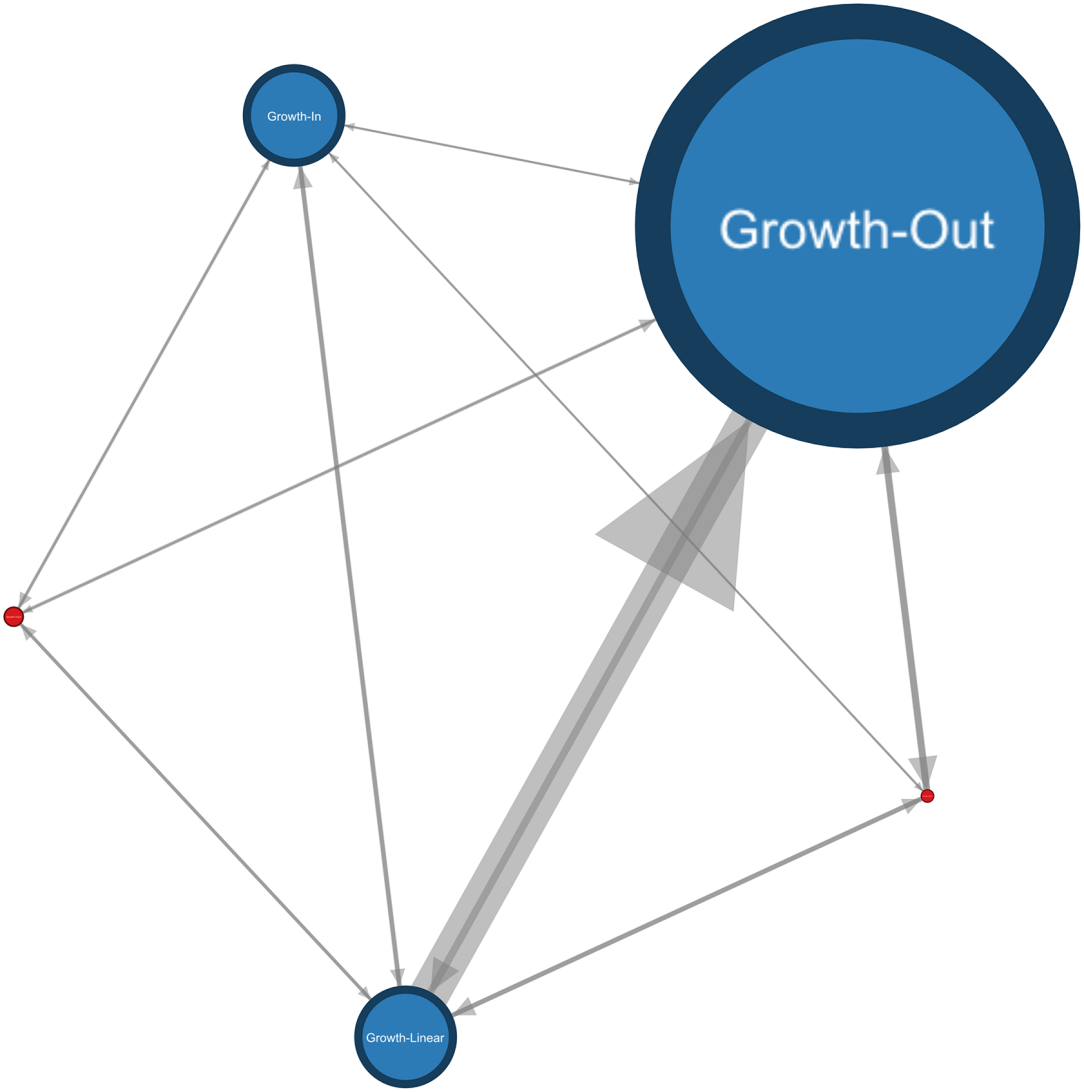
Observed lifecycles in ArxivTH. The size of the nodes is proportional to the trend’s fractions in the network by the end of the dataset. The nodes in red are the Growth-InOut on the left and Growth-OutIn trend on the right. The size of the edges is proportional to the number of observed transitions.

The fundamental question to be answered in this paper goes as follows: “If an article starts to suffer a loss of citations, this probably indicates a transfer of attention to another publication. Is it possible to identify the beneficial papers? Are there any clear types of transfer occuring?”. The most interesting papers to examine are the ones showing a Growth-Out type. Among the different datasets used, those do represent a significant proportion (e.g: More than 60% of the article having more than 10 citations in Arxiv and APS are Growth-Out. For PubMed, more than 60% before 2004 but only 20% by the end of 2012. This difference is mainly due to a major shift in the way PubMed indexed external sources of information after this date.) The Fig 7 summarizes all possible transition patterns between the growth trends identified in ArxivTH as well as the fractions of each trend by the end of the dataset. As indicated, the most frequent transitions go to Growth-Out. Less common transitions like ‘Growth-In to Growth-Out’ may be observed even though the intuition would suggest the following smoother transition: Growth-In to Growth-Linear to Growth-Out. This is due to some young articles not presenting enough data soon after their publication and leading to major shifts in their trend where most of the articles will cover a longer period of time and thus present smoother transitions overall.

The algorithm to be presented in the following sections focuses on the construction of genealogical trees whose starting nodes are of the Growth-Out type, indicating that they already lost most of their popularity. A dynamical visualisation of each tree being built is possible but beyond the scope of the hereby-presented work. Here, only static trees are presented showing the entire life of the starting node together with the offspring that take over the popularity of their parents.

## The generation of genealogical trees

Our whole analysis should be perceived through some sort of evolutionary lenses driving to the construction of genealogical trees answering this question: ‘What could be the descendants of an article after its demise?’ The definition of such trees slightly differs from the conception of genealogical trees in real life. The children of an article are the articles that cite it and thus inherit something from it, explaining why the citation was done in the first place.

The construction and presentation of such trees does however present the following challenge. Let’s consider a prominent article from the Arxiv TH: 9711200, an article that received more than a thousand citations. If a tree had to be built, this would lead to one thousand children at the first level (i.e: Direct citation from a child to its parent) and many more at the second level (i.e: Citation to the parent from a great child through their own parents). The density and complexity of the resulting graph will hamper its readability. As a solution, we propose an algorithm that uses the properties of the four growth trends previously discussed together with a ranking method in order to select a small subset of offspring.

### Algorithm

The algorithm (Algorithm 1) used to construct our genealogical trees is recursive depth-first, applying at each step of the construction a ranking-based selection of the most prominent articles. A few hypothesis are still necessary to avoid overloading any tree with too much information and compromising its readability.

1. Any article appears only once in a given tree. Although a same article could be the descendant of several nodes distributed wherever in the tree, only its first appearance will be conserved in the resulting tree. This may however be set differently in Algorihm 1 at line 8.
2. The algorithm must be able to pinpoint the loss of popularity of articles that are either Growth-Linear or Growth-In. To keep the method simple and robust, the points in time it looks for are the crossing point between *q*2 and *q*3 or *q*5. (i.e: Growth-In article becoming Growth-InOut or Growth-Linear article becoming Growth-Out). This tendency must also be preserved for a few months to discard the possibility of a simple glitch in the dataset on account of the low time resolution being used.
3. Only the five most prominent articles are conserved as offspring. This may be set at line 8 of Algorithm 1.

**Algorithm 1**: Genealogical tree generation algorithm

 **Data**: ID of the article, DATEFINAL at which to produce the tree, LEVEL of recursivity

 **Result**: Genealogocial tree of the article with ID

1 ARTICLE with ID is retrieved

2 DATECURRENT is initialized at DATEPUBLICATION of article with ID

3 CHILDREN is initialized as an empty array

4 DATECROSSING is computed by analyzing the quadrant values of article with ID

5 **while** *DATECROSSING* < *DATEFINAL* **do**

6  read citations received by the article at DATECROSSING and push them to CHILDREN

7  compute the ranking of the CHILDREN

8  **for all** *CHILD in CHILDREN not already in tree* **do**

9   call ALGORITHM with CHILD, DATECROSSING and LEVEL-1 as parameters

10  **end**

11 **end**

Finally, in the figures to be presented below, the size of each node in a tree is proportional to the score of the ranking at the time of the crossing that was detected. The colour of each node in a tree represents the type of growth of the article; blue for the Growth-Out, green for the Growth-In and different shades between green and blue for the others. Orange nodes are the ones that display a Growth-InOut trend at the indicated time, this was done to emphasize on their particular behaviour.

In substance, at each step, the algorithm searches for a crossing point among the quadrants time series of the given article, a *crossing point* being the point in time where two quadrant curves do cross each other. More specifically, our algorithm searches for crossing between *q*2 and *q*3 or *q*5 values, thus identifying moments where the popularity of an article clearly shifts. The algorithm then continues by recursively repeating the same operation on each selected child and selected great child. The ranking algorithm we used is our own, but could be any of the other existing ones like PageRank [14], CiteRank [15], FutureRank [16] or others. Our ranking algorithm improves on existing ones in emphasizing more recent articles and not requiring the knowledge of the entire network to be computed. By only taking into account the trend followed by each article growth curve, and not the entire network topology, it can be applied in a dynamical way, allowing to observe in real-time the tree being built. Using only the quadrant values for each article, this algorithm tries to anticipate the future popularity of any paper and puts more emphasis on the young and promising ones. This ranking method allows to limit the number of children to only five, thus keeping the generated trees small and readable.

## Results

Below we present clear examples of old and once quite popular articles but having almost totally lost their popularity at the time each tree is built, so articles of the Growth-Out types.

### Example on ArXiV TH

**Fig 8:**
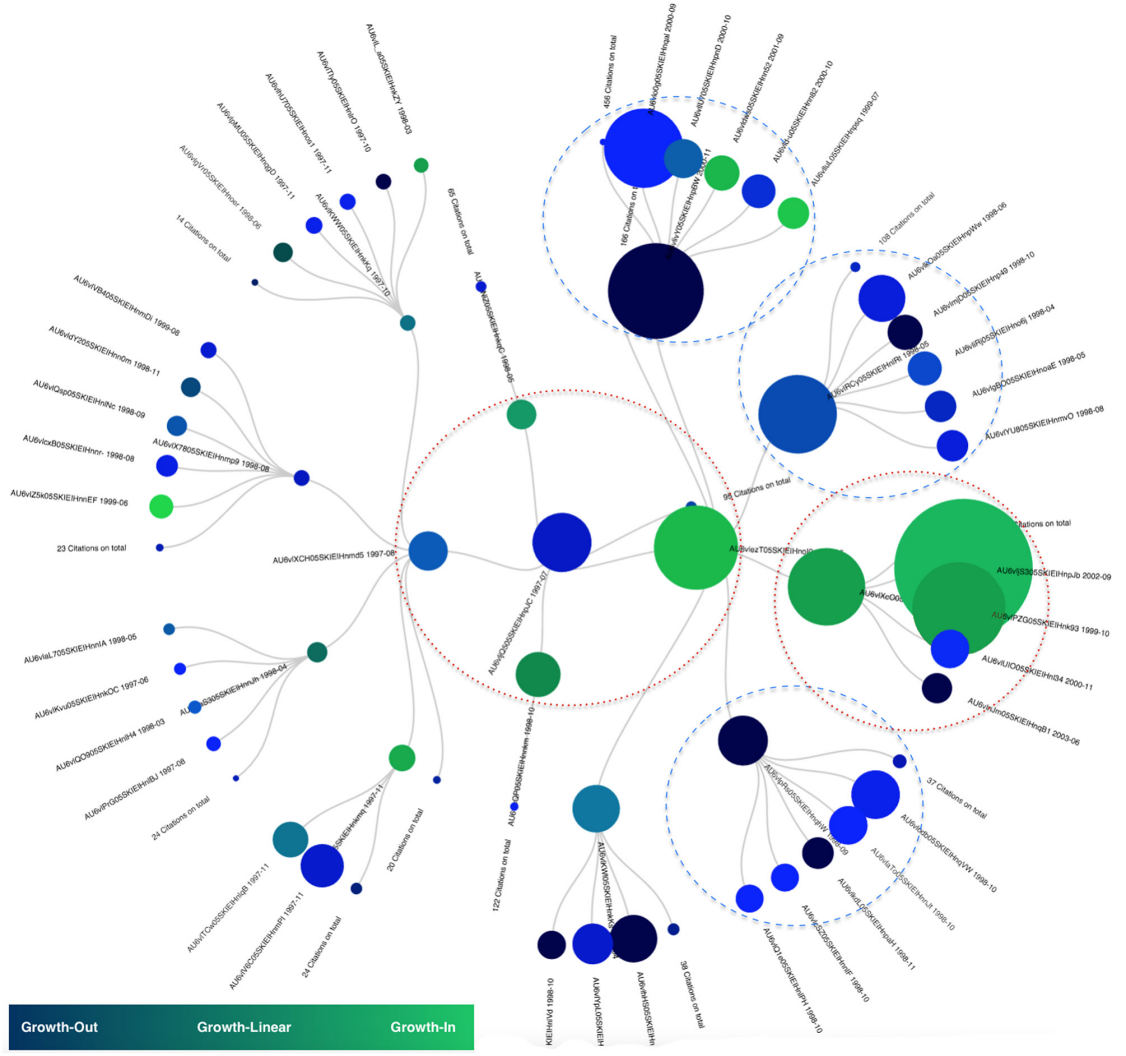
Genealogocial tree of article AU6vljQ505SKIEIHnpJC.

The genealogical tree of the article AU6vljQ505SKIEIHnpJC is presented in Fig 8. This article, entitled “Notes on D-Branes”, was published in February 1996 by Polchinski, Chaudhuri and Johnson. At the moment it started losing popularity, around July 1997, the most prominent articles were AU6vlezT05SKIEIHnoI0, AU6vlRCy05SKIEIHnlRt and AU6vlcQP05SKIEIHnnkm. “TASI Lectures on D-Branes” (i.e: AU6vlRCy05SKIEIHnlRt) was a new article by Polchinski that supposedly went further than “Notes on D-Branes” on the same topic and was naturally stealing the attention from its parent. The other offspring are: “M Theory As A Matrix Model: A Conjecture” (i.e: AU6vlezT05SKIEIHnoI0) published by Banks, Fischler, Schenker and Susskind and “D-Branes and Short Distances in String Theory” (The title clearly testifies of the continuity of the topic) published by Doublas, Kabat, Poulliot and Schenker. Those two articles did gather a lot of interest too and equally stole the light out of their parent.

During the following years two other articles were published and continued draining more and more light from “Notes on D-Branes”. “The Large N Limit of Superconformal Field Theories and Supergravity” (i.e: AU6vlXcO05SKIEIHnmjQ) published by Maldacena and “Anti De Sitter Space and Holography” published by Witten were two great children that also contributed to the loss of popularity of their parent.

### Example on APS

**Fig 9:**
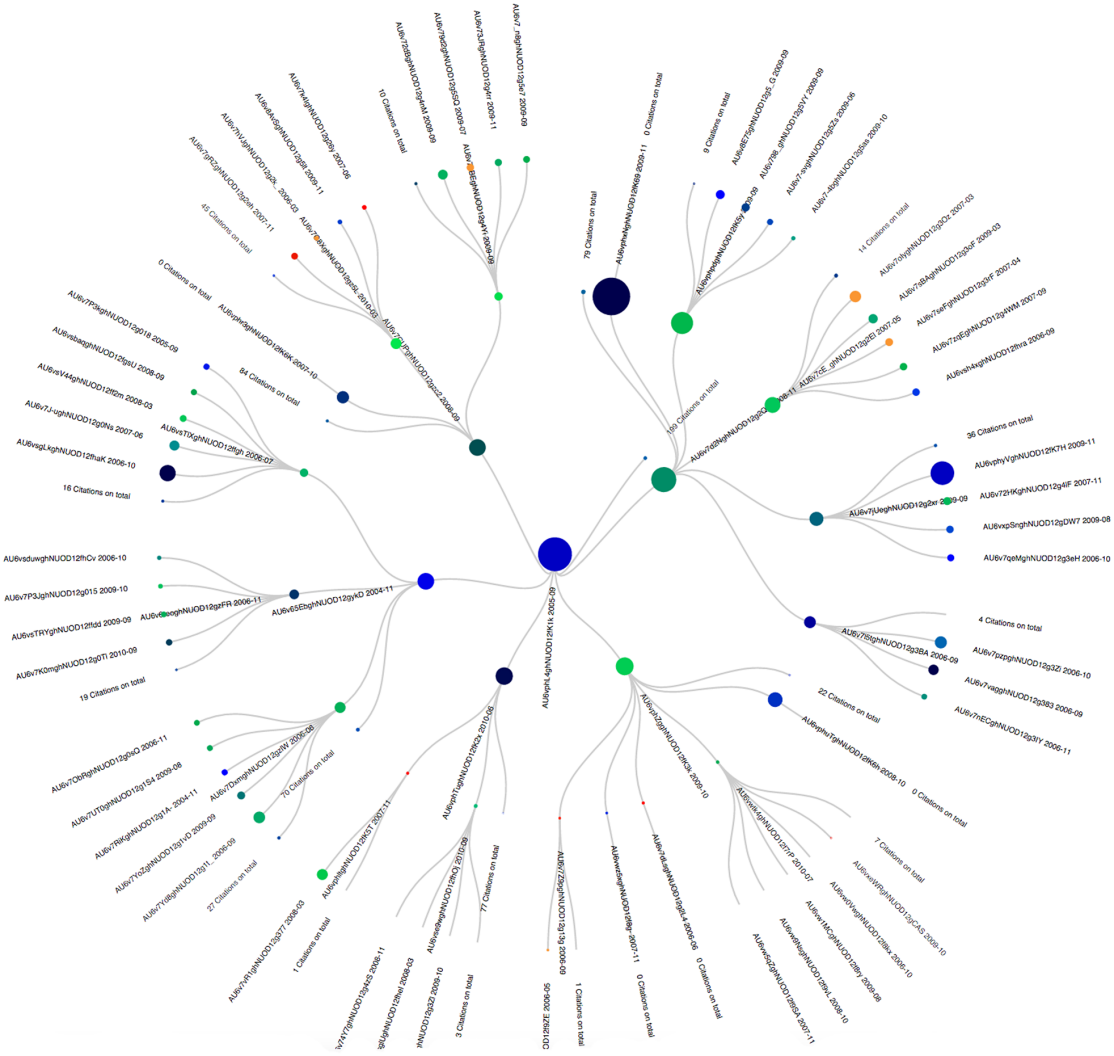
Genealogocial tree of article AU6vphL4ghNUOD12fK1k.

The article “Theory of Bose-Einstein condensation in trapped gases” by Dalfovo, Giorgini, Pitaevskii and Stringari was published in April 1999 (i.e: AU6vphL4ghNUOD12fK1k). Its genealogical tree is presented in Fig 9. This paper started losing popularity around September 2005. Looking at the situation back then, three articles were taking the front of the scene: “Quasipure Bose-Einstein Condensate Immersed in a Fermi Sea” (i.e: AU6v7FkaghNUOD12gzwO), “Vortex Formation in a Stirred Bose-Einstein Condensate” (i.e: AU6v65EbghNUOD12gykD) and “Bose-Einstein condensation in the alkali gases: Some fundamental concepts” (i.e: AU6vphTughNUOD12fK2x). In all three cases again, the title clearly indicates the scientific content continuity.

### Example on Pubmed

**Fig 10:**
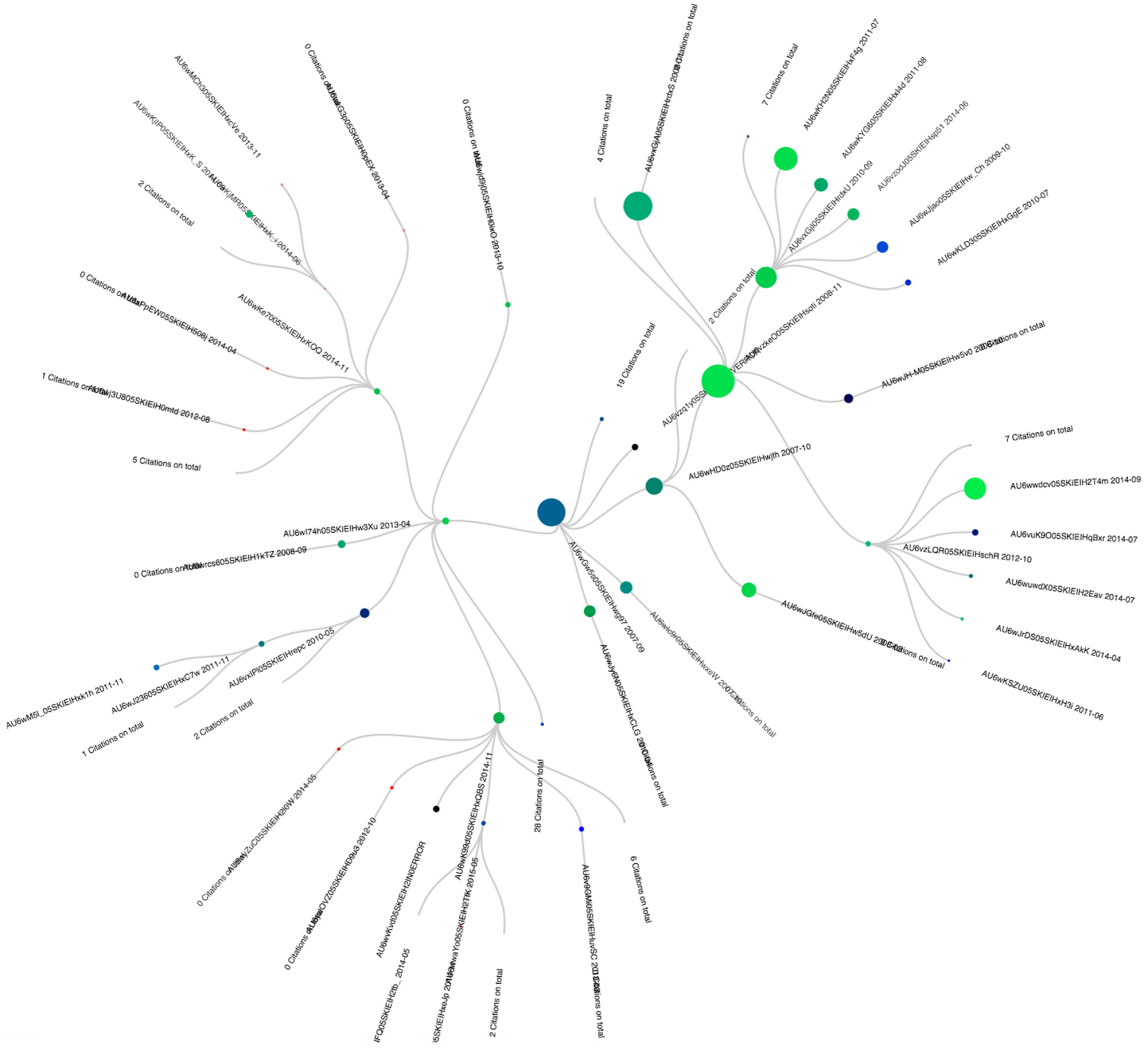
Genealogocial tree of article AU6wGw5s05SKIEIHwg97.

The starting node of the genealogical tree presented in Fig 10 is entitled “The Pfam protein families database”. It was published in October 2003 and was written by Bateman, Coin, Durbin Finn, Hoolich, Griffiths-Jones et al. The different children present in the hierarchy are either follow ups of this initial publication like “Pfam: clans, web tools and services”” or novel proposals like “Ensembl 2006”, “Ensembl 2007”, “Ensembl 2008” and “The Universal Protein Resource (UniProt): an expanding universe of protein information” respectively. All those papers do talk about databases and their updates overtime, an aspect that obviously explains how and why they gradually loose interest to the profit of more recent publications.

## Conclusion

We have shown three examples of genealogical trees constructed on the basis of different scientific citation networks. All highlighted articles are quite well known in their own field. Those examples do illustrate the most common ways one article may lose citations to the profit of others. Essentially, two frequent paths of evolution may be followed by popular articles.

First, a paper may have been a star and have become a reference in its domain, remaining as such for a long time [22]; it still gathers references as time passes even though this gathering rate diminishes with time. Those articles may be compared to the ones firstly arrived in the network and profiting from the “First-Mover Effect” described by Newman [23]. In such cases, no offspring may really be distinguished and be pointed out as potential new stars ready to steal the show. The parent stays the most referenced and keeps a long lasting influence even though its initial pace of growth decreases with time [22] (Examples on Fig 8 circled in a blue dashed line).

Second, an article may suffer from a loss of citations because a novel paper, more remarkable and appealing, was published with obvious consequence to capture the attention initially focused on its parent. Those examples are the most frequent. Some are illustrated on Fig 8 circled in red dots. Those articles give rise to children that do gather more attention than they used to i.e a higher growth rate as well as a Growth-In or Growth-Linear trend. This growth rate in some cases may be twice the one of the parent or more and such papers can be referred as the new stars [24] of the network. “Sleeping Beauties” [25] may as well appear as new stars of the network even though it may take longer for them to shine in their genealogy. However, restricting this genealogy only to static trees make such papers very difficult to appear. This bottleneck may be simply avoided by relying on a dynamical version of the algorithm in which the ranking and the most prevalent offspring would be updated as time passes. This dynamical version will be presented in future works, together with a novel typology of popularity transfer among articles. We are right now more closely observing the dynamical citations practices and trying to understand better the sociological phenomena ruling the growth of such networks.

We also incidentally show in this paper (a coming publication will be entirely dedicated to this topic) how to make use of ranking algorithms in a new way compared to the usual practises. Many different ranking algorithms [14] [15] [16] do exist as well as lots of variations of these original counterparts. They are however almost always applied in a static fashion, implying that a ranking is computed at only one precise timestamp. Comparison are then made between two rankings established early in the network then later on, to try making predictions [16] [26] on the future ranking or citation count of an article. Moreover, those rankings are usually computed globally, at the scale of an entire dataset, which would make the algorithm presented here much too slow for efficiently building the genealogical trees. We thus rely on a different kind of ranking that offers similar results to the existing alternatives yet using much less information and not requiring the computation over the entire network.

This paper proposes a study of scientific articles, their success and fate, in a sort of evolutionary framework. In our proposal, the fitness of an article in this very dense jungle of publications, amounts to its rank and its descendants are assimilated to the more recent and most successful articles quoting it, thus propagating their “DNA” content with new variations. Very practically, departing from a key article in a scientific field, our method might allow any researcher in this specific field to guide his exploration by tracing the most important steps and bifurcations that lead to the current state of the art.

## References

[1] Price D.J. de Solla. Networks of Scientific Papers. Science, 149, pp 510–515, 1965 10.1126/science.149.3683.510 14325149

[2] Price D.J. de Solla. A general theory of bibliometric and other cumulative advantage processes. Journal of the American Society for Information Science, 27, pp 292–306, 1976 10.1002/asi.4630270505

[3] Redner S. Citation statistics from 110 years of physical review. Phys. Today 58, 49, 2005 10.1063/1.1996475

[4] Barabasi A.-L and Albert R. Emergence of scaling in random networks. Science, 286, pp 509–512, 1999 10.1126/science.286.5439.509 10521342

[5] Newman M.E.J. The structure of scientific collaboration networks. PNAS Vol.98 No2, pp 404–409, 7 2000 10.1073/pnas.98.2.404 PMC14598 11149952

[6] Pan R.K, Saramaki J. The strength of strong ties in scientific collaboration networks. Europhys. Lett. 97, 18007, 6 2011 10.1209/0295-5075/97/18007

[7] Pepe A. Structure and Evolution of Scientific Collaboration Networks in a Modern Research Collaboratory. 5 2010 10.2139/ssrn.1616935

[8] Eom Y-H, Fortunato S. Characterizing and Modeling Citation Dynamics. 9 2011 10.1371/journal.pone.0024926 PMC3178574 21966387

[9] Medo M, Cimini G, Gualdi S. Temporal effects in the growth of networks. Physical Review Letters 107, 238701, 9 2011 10.1103/PhysRevLett.107.238701 22182132

[10] Hirsch J.E. An index to quantify an individual’s scientific research output. PNAS, Vol 102, No 46, pp 16669–16572, 11 2005 10.1073/pnas.0507655102 PMC1283832 16275915

[11] Batista P.D, Campiteli M.G, Kinouchi O. Is it possible to compare researchers with different scientific interests? Scientometrics 68 (1), pp 179–189, 7 2006 10.1007/s11192-006-0090-4

[12] Sidiropoulos A, Katsaros D, Manolopoulos Y. Generalized Hirsch h-index for disclosing latent facts in citation networks, Scientometrics 72 (2), pp 253–280, 6 2007 10.1007/s11192-007-1722-z

[13] Watts D.J, Strogatz S. Collective dynamics of’small-world’ networks, Nature 393 (6684), pp 440–442, 6 1998 10.1038/30918 9623998

[14] Brin S, Page L. The Anatomy of a Large-Scale Hypertextual Web Search Engine. Computer Networks and ISDN Systems, Vol. 30, Issue 1-7, pp 107–117, 4 1998 10.1016/S0169-7552(98)00110-X

[15] Walker D, Xie H, Yan K-K, Maslov S, Ranking Scientific Publications Using a Simple Model of Network Traffic, J.Stat.Mech. 0706 p06010, 2007 10.1088/1742-5468/2007/06/P06010

[16] Sayyadi H, Getoor L. FutureRank: Ranking Scientific Articles by Predicting their Future PageRank. SIAM International Conference on Data Mining SDM09, 2009 10.1137/1.9781611972795.46

[17] Yao L, Wei T, Zeng A, Fan Y, Di Z. Ranking scientific publications: the effect of nonlinearity. Scientific Reports 4, Art:6683, 5 2014 10.1038/srep06663 25322852 PMC4200399

[18] Ghosh R, Kuo T-T, Hsu C-N, Lin S-D, Lerman K. Time-aware Ranking in Dynamic Citation Networks. pp 373–380, 12 2011 10.1109/ICDMW.2011.183

[19] Krapivin M, Marchese M. Focused Page Rank in Scientific Papers Ranking. pp 144–153, 2008 10.1007/978-3-540-89533-6_15

[20] Valverde S, Sola R.V, Bedau M.A, Packard N. Topology and evolution of technology innovation networks. Phys. Rev. E 76, 056118, 28 11 2007 10.1103/PhysRevE.76.056118 18233729

[21] Penner R. Programming Macromedia Flash MX. Osborne 2002, Part 3, Dynamic Visuals, ISBN13:978-0072223569, ISBN10:0072223561, http://robertpenner.com/easing/

[22] Wang D, Song C, Barabasi A-L. Quantifying Long-Term Scientific Impact. 10 2013 10.1126/science.1237825 24092745

[23] Newman M.E.J. The first-mover advantage in scientific publication. EPL, Vol. 86, pp 68001 p1–6, 6 2009 10.1209/0295-5075/86/68001

[24] Li X-L, Foo C.S, Tew K.L, Ng S-K. Searching for Rising Stars in Bibliography Networks, Database Systems for Advanced Applications, Volume 5463, pp 288–292, 2009 10.1007/978-3-642-00887-0_25

[25] Ke Q, Ferrara E, Radicchi F, Flammini A. Defining and identifying Sleeping Beauties in science. Proc. Natl. Acad. Sci. USA 16, pp 7426–7431. 6 2015 10.1073/pnas.1424329112 PMC4475978 26015563

[26] Newman M.E.J. Prediction of highly cited papers. Europhys. Lett. 105, pp 28002, 2014 10.1209/0295-5075/105/28002

